# Clinical and Microbiological Effects of Weekly Supragingival Irrigation with Aerosolized 0.5% Hydrogen Peroxide and Formation of Cavitation Bubbles in Gingival Tissues after This Irrigation: A Six-Month Randomized Clinical Trial

**DOI:** 10.1155/2020/3852431

**Published:** 2020-07-31

**Authors:** Gediminas Žekonis, Renata Šadzevičienė, Ingrida Balnytė, Viktorija Noreikienė, Gaida Marija Šidlauskaitė, Eglė Šadzevičiūtė, Jonas Žekonis

**Affiliations:** ^1^Department of Dental and Maxillofacial Orthopedics, Medical Academy, Lithuanian University of Health Sciences, Kaunas, Lithuania; ^2^Department of Dental and Oral Pathology, Medical Academy, Lithuanian University of Health Sciences, Kaunas, Lithuania; ^3^Department of Histology and Embryology, Lithuanian University of Health Sciences, Kaunas, Lithuania; ^4^Joint Stock Company “MediCA Klinika”, Lithuania

## Abstract

**Introduction:**

The study investigated the effect of weekly supragingival irrigation with aerosolized 0.5% hydrogen peroxide (H_2_O_2_) solution as a maintenance periodontal therapy on clinical and microbiological parameters in patients with chronic periodontitis. The other purpose was to investigate whether cavitation bubbles can penetrate not only into periodontitis-damaged tissues but also into *ex vivo* porcine healthy periodontal tissues.

**Materials and Methods:**

The study included 35 systemically healthy patients with chronic periodontitis (CP). After nonsurgical periodontal debridement (NSPD), all patients were randomized into two groups: the Control group (NSDP alone, *n* = 18) and the Test group (NSDP plus supragingival irrigation, *n* = 17). Clinical (Approximal Plaque Index (API), Bleeding Index (BI), and Modified Gingival Index (MGI)) and microbiological (Polymerase Chain Reaction technology (using a micro-IDent® kit)) measurements were performed at the initial time point, 3 months, and 6 months after NSPD. The impact of supragingival irrigation on diseased gingival tissues of CP patients (*n* = 5) and on *ex vivo* porcine healthy gingival tissue samples (*n* = 3) was evaluated to estimate morphological changes in healthy and diseased gingival tissues.

**Results:**

Morphological data revealed that supragingival irrigation caused the formation of cavitation bubbles in diseased gingival tissue of CP patients and in healthy porcine gingival tissues. The decrease in API, BI, and MGI scores after 6 months in the Test group significantly (*p* ≤ 0.01, *p* ≤ 0.05, and *p* ≤ 0.01, respectively) exceeded that in the Control group. Test group patients demonstrated a decrease in periodontal sites showing Pocket Probing Depth > 4 mm and, after 6 months, a statistically significant decrease in the proportion of periopathogenic bacteria.

**Conclusion:**

The effectiveness of mechanical periodontal treatment combined with weekly supragingival irrigation with aerosolized 0.5% H_2_O_2_ solution on clinical and microbiological parameters of periodontal tissues of periodontitis patients is reliably higher than that of mechanical periodontal debridement alone. It has been found that cavitation bubbles as a result of irrigation with the aerosolized 0.5% hydrogen peroxide solution can form not only in periodontal tissues of periodontitis patients but also in *ex vivo* porcine healthy gingival tissues.

## 1. Introduction

Periopathogenic species seem to play a key role in the etiology and pathogenesis of periodontal diseases [1]. The main bacteria that cause periodontitis are the following: *Aggregatibacter actinomycetemcomitans* (Aa), *Porphyromonas gingivalis* (Pg), *Tannerella forsythia* (Tf), *Treponema denticola* (Td), and *Prevotella intermedia* (Pi) [2]. However, recent research shows that while bacteria are necessary for periodontitis development, they are not sufficient for the clinical manifestation of this disease [3]. It is becoming obvious that it is the host immune response to the periopathogenic species that is responsible for the progression of the disease and tissue damage [1, 4].

Many researches have pointed to the role of genes and their variants in host response and susceptibility to periodontitis. Since at the present it is impossible to revise the susceptibility of the host to periodontitis [4], the principle of therapy of periodontal tissues is the killing or reduction of periopathogenic microorganisms [5]. Mechanical cleaning of the tooth and root surfaces is the key stone of the conventional periodontal treatment, which destroys the biofilm and reduces bacterial accumulation [6]. These treatment procedures reduce the amount and proportion of periodontal pathogens and prevent further damage of the periodontal tissues [7]. Various treatment methods, including lasers [8], photodynamic therapy [9], and different antimicrobial agents, have been suggested as additional therapeutic measures for the reduction of the number of bacterial colonies. Some of these adjunctive measures show good clinical results; however, overall microbiological improvement is still insufficient [10]. The alarming worldwide increase in antibiotic resistance among microbial pathogens necessitates a search for new antimicrobial techniques [11].

Research has begun to evaluate the use of low-cost antimicrobial agents as additions to conventional periodontal therapy [12, 13]. In our recent work, we have successfully tested hydrogen peroxide (H_2_O_2_) [14]. H_2_O_2_ has been used for a long time as an effective antimicrobial agent with a broad spectrum of activity (including bacterial spores and viruses) [15] and is widely used in cases where it is important to decompose it into nontoxic by-products (water and oxygen) [16]. Recently, it is shown that in oral biofilms, commensal species suppress the amount of pathobionts by H_2_O_2_ production [17]. Literature indicates and our recent study can confirm that a sprayer for liquid aerosolization is a good method for the delivery of H_2_O_2_ into the subgingival pocket [14, 18]. At the same time, good results can be achieved by using lower drug concentrations [18]. Aerosolized H_2_O_2_ has also been used to effectively eliminate microorganisms from surfaces [19], and the bactericidal efficacy of aerosolized H_2_O_2_ exceeds the effects of pure H_2_O_2_ solution [15]. A superior bactericidal effectiveness of aerosolized H_2_O_2_ may be explained by the fact that due to the hydrodynamic cavitation phenomenon, strong turbulence of the H_2_O_2_ solution in the nozzle hole of the spray contributes greatly to the disintegration of the liquid jet and the formation of cavitation bubbles [20]. These bubbles are known to form following acoustic and hydrodynamic cavitation [21, 22]. Cavitation is the formation, growth, and collapse of gas- or vapor-filled bubbles within a liquid medium due to local pressure variation [23]. The quick collapse of these bubbles can cause very high temperature and pressure inside them [24]. Due to such severe conditions, the molecules inside the bubbles can be brought into an excited state and dissociate. Various reactive oxygen species (ROS) can be created from H_2_O, O_2_ and H_2_O_2_ dissociation and their associate reactions inside the bubble [25]. It is known that ^·^OH has a much higher reactivity and oxidative power than H_2_O_2_ [26]. Consequently, the abovementioned phenomenon might clarify an increased bactericidal efficacy of aerosolized H_2_O_2_ solutions. The data of our recent study demonstrate that supragingival irrigation with aerosolized H_2_O_2_ solution can create cavitation bubbles which penetrate into periodontal tissues and may increase bactericidal effects of aerosolized H_2_O_2_ [14].

However, the observation of cavitation bubbles deep within tissues is very difficult [27] because they have a very short life span [28]. Literature in this field is scarce, probably due to the complicated nature of the phenomenon [25].

Thus, the future of supragingival irrigation is promising, and it should provide a more predictable adjunct for the treatment and maintenance of periodontitis patients [14, 29]. However, whether this intervention may have an impact on periodontal parameters still needs to be assessed [14, 30].

Based on the abovementioned literature data, the aim of this randomized study was to update the results of our previous research concerning the impact of regular supragingival irrigation with aerosolized H_2_O_2_ solution as a supportive periodontal therapy on clinical and microbiological parameters of periodontal tissues of periodontitis patients.

The other purpose was to investigate whether cavitation bubbles can penetrate not only into periodontitis-damaged tissues but also into *ex vivo* porcine healthy periodontal tissues.

## 2. Materials and Methods

### 2.1. Patient Selection

In total, 39 untreated adult patients with chronic periodontitis were selected for this study from new patients attending to the Faculty of Odontology of the Lithuanian University of Health Sciences. Screening examinations included medical history and clinical examinations.

The following *inclusion criteria* were used for participation in this prospective longitudinal study:

- 20-50 years of age
- Minimum of 20 teeth in the mouth
- Each patient had at least two nonadjacent sites per quadrant with the Pocket Probing Depth (PPD) of at least 5 mm, which bled upon probing
- Each patient had radiographic evidence of horizontal and vertical bone loss

*Conditions leading to the exclusion from the study* were as follows:

- Systemic disorders (diabetes mellitus, rheumatoid arthritis, or osteoporosis)
- Systemic use of antibiotics during the study or 4 months before study
- Smoking
- Alcohol intake

Prior to the beginning of the study, a decision was made that subjects who exhibited a marked disease progression (inflammation or suppuration) should be excluded from the trial and should receive additional periodontal treatment.

### 2.2. Study Outline

#### 2.2.1. Randomization

After the first screening visit, all patients were listed alphabetically and then sequentially assigned: one patient to the Test group, the other to the Control group, and so on (Control group: 18 patients; Test group: 21 patients). The sample size was calculated after performed study power calculation.

Following the completion of the nonsurgical periodontal debridement (NSPD) procedure, all patients were assigned for oral hygiene instruction. One week after NSPD, Test group subjects underwent weekly supragingival irrigation with 0.5% aerosolized H_2_O_2_. To ensure blindness, one investigator performed NSDP and another investigator collected clinical data on the Control and Test groups. To emphasize the influence of weekly supragingival irrigation on periodontal tissues, the patients were asked not to alter their usual lifestyle and diet.

Four patients of the Test group dropped out from the study because of irregular attendance. All patients in this study signed the informed consent form approved by Kaunas Regional Bioethics Committee. The research was performed in accordance with the rules and regulations approved by Kaunas Regional Bioethics Committee (No. BE-2-21).

### 2.3. Treatment Procedure Using Irrigation

Treatment procedure was performed according to Žekonis et al. (2016). For the supragingival irrigation procedure, the dental unit Knight Asepsis (Midmark Corp., Versailles, OH, USA) angular air and water dental syringe was used along with a reservoir for hydrogen peroxide solution, which could be adjusted for the pressure of about 80 psi and water outflow of 100 mL/min [14]. We selected 0.5% concentration of hydrogen peroxide because irrigation with higher concentrations of the aerosolized H_2_O_2_ solution caused gingival pain in the pilot study patients. The solution (200 mL) was applied with the irrigator on the gingival margins at a 45-degree angle [29]. The temperature of the hydrogen peroxide used during the procedure was 37°C. Each irrigation procedure required approximately 5 min to use up the entire solution and irrigate all the teeth [14]. The assessment of the effect of supragingival irrigation on the clinical and microbiological indices was then repeated after 3 and 6 months.

### 2.4. Clinical Measurements

Four periodontal indices were used in the study.

#### 2.4.1. Approximal Plaque Index (API)

Oral hygiene was assessed using API according to Lange et al. (1977). The total percentage of plaque-containing surfaces was calculated [31].

#### 2.4.2. Modified Gingival Index (MGI)

To assess gingival inflammation, the MGI (proposed by Lobene et al. (1986)) was calculated on the facial and lingual surfaces at two sites of each tooth (the papillae and the margin) [32]. These assessments were performed on all the evaluated teeth. Compressed air, water, and mouth mirrors were available to the examiner. The mean MGI was calculated by dividing the sum of all scores by the total number of the examined surfaces.

#### 2.4.3. Bleeding Index (BI)

The Bleeding Index (a modified Index of Saxton (1989)) was determined using the Williams periodontal probe in the evaluation (Hu-Friedy, Chicago, IL, USA) [33]. The number of sites where bleeding is recorded is divided by the total number of available sites in the mouth and multiplied by 100 to express the BI.

#### 2.4.4. Pocket Probing Depth (PPD)

Pocket Probing Depth (PPD) was measured and recorded at six sites per tooth (distobuccal, buccal, mesiobuccal, mesiolingual, lingual, and distolingual) using the Williams periodontal probe. The examiner was trained and calibrated in performing the clinical measurements before the examinations. The procedures were performed manually by a single experienced periodontist (R.S.).

### 2.5. Microbiological Measurements

The assessment of the microbiological impact of long-term supragingival irrigation with aerosolized 0.5% H_2_O_2_ solution was performed using a micro-IDent® kit (Hain Lifescience GmbH, Nehren, Germany).

#### 2.5.1. Collection of Microbial Samples (according to Cosyn and Sabzevar (2007))

At initial time point (prior to NSPD and supragingival irrigation) and at 3 and 6 months (also before supragingival irrigation), subgingival microbial samples were taken by the same investigator (R.S.) from the deepest pocket per quadrant. The samples were taken from the same sites on all time points. Each selected site was dried and isolated from saliva using cotton rolls. Subsequently, sterile PerioPaper Strips (Oraflow Inc., Hewlett, NY, USA) was inserted and left in place for 20 seconds. Four samples per patient were collected and sent to the laboratory for analysis [5].

#### 2.5.2. Polymerase Chain Reaction (PCR)

PCR technology (using a micro-IDent® kit) was used to identify Aa, Pg, Tf, Td, and Pi according to Eick and Pfister [34].

Bacterial levels were expressed as genome equivalents (10^3^ to 10^4^—low bacterial level and 10^5^ to 10^6^—high bacterial level). The test had a detection limit of 10^3^ genome equivalents.

### 2.6. Morphological Findings of Periodontal Tissues

If areas with inflammation and PPD ≥ 5 mm were found in the 6-month time point, immediately after supragingival irrigation, gingivectomy was performed under local anesthesia. The specimens of the resected gingival tissues were fixed in 10% neutral-buffered formalin, embedded in paraffin, sectioned in 3 *μ*m sections, and then stained with hematoxylin and eosin (HE), according to the standard staining protocol.

The evaluation of the impact of supragingival irrigation on diseased gingival tissues of patients with CP (*n* = 5) and on experimental *ex vivo* porcine healthy gingival tissues (*n* = 3) was performed morphologically.

To estimate the impact of supragingival irrigation on healthy gingival tissues, 3 lower jaws of slaughtered porcine were obtained and stored at -20°C prior to the experiment. On the day of the experiment, the porcine jaws were defrosted in physiological saline at 37°C. The irrigation procedure and the evaluation of the impact of this procedure on porcine healthy gingival tissues were performed as describe above. Nonirrigated gingival tissues of patients with CP and *ex vivo* porcine served as control samples. This histological evaluation of the samples was performed with a cold light microscope Olympus BX53 (Olympus Corporation, Tokyo, Japan) under 4x, 10x, and 20x magnification using digital processing software Image-Pro® Plus 7.0 (Media Cybernetics, Inc., Bethesda, MD, USA).

### 2.7. Reagents

Hydrogen peroxide (30% analytical grade) was obtained from Reachem (Bratislava, Slovakia). A 6% stock solution was prepared in distilled water. The 0.5% H_2_O_2_ solution for supragingival irrigation was prepared daily. Formalin 30% neutral-buffered was obtained from Sigma-Aldrich (St. Louis, MO, USA).

### 2.8. Statistical Analysis

Statistical analysis of the data was performed by using the statistical software package SPSS 20.0 for Windows (SPSS, Inc., Chicago, IL, USA). All parametric data were expressed as mean and standard deviation (M (SD)). The Mann–Whitney *U* test was used to compare quantitative sizes of two independent samples. Wilcoxon nonparametric test was used for quantitative dependent data. Chi-square (*χ*^2^) tests were used to compare frequencies of qualitative variables. A *p* value of 0.05 or less was considered significant.

## 3. Results

### 3.1. Clinical Measurements

A total of 39 randomized patients with chronic periodontitis were screened for their eligibility to participate in this controlled study (to attend examinations for 6 months). Dropouts (four in the Test group) were due to lack of compliance. The remaining 35 systemically healthy patients (18 in the Control group and 17 in the Test group) completed the study. The distribution of the sampled sites (Table 1) was equal in both groups according to their origin.

Table 1 shows the initial time point data of the patients in the Control and Test groups. At this time point parameters were similar in both groups (*p* > 0.05). All subjects completed the six-month study period and met the requirements of the study. No side effects were observed during this period.

The mean API, MGI, BI, and PPD values for the initial, 3-month, and 6-month time points for both groups are presented in Table 2. The initial time point API and BI scores in the Control group were reduced (*p* ≤ 0.05 and *p* ≤ 0.01, respectively) at month 3 and remained relatively low during the whole study. Meanwhile, the initial time point API, BI, and MGI scores of the Test group were markedly (*p* ≤ 0.05, *p* ≤ 0.01, and *p* ≤ 0.05, respectively) reduced at month 3 and particularly at month 6. These decreases exceeded (*p* ≤ 0.01, *p* ≤ 0.05, and *p* ≤ 0.01, respectively) the analogous data of the periodontitis patients in the Control group. A significant (*p* ≤ 0.05) reduction in PPD was observed only in the Test group (Table 2). It is noteworthy that there was a decrease (*p* ≤ 0.05) in the proportion of periodontal sites showing PPD > 4 mm in the Test group at month 6 following weekly supragingival irrigation with aerosolized 0.5% H_2_O_2_.

### 3.2. Microbiological Evaluation

The mean values of obligate anaerobes (%) at the initial time point and at months 3 and 6 for both groups are presented in Table 3. The proportion of sites that were positive for several bacteria decreased. A statistically significant (*p* ≤ 0.05) decrease in Td-, Tf-, and Pg-positive sites was detected only in the Test group after 6 months. Other microbiological results expressed as a positive bacterial sample per site were nonsignificant.

**Figure 1:**
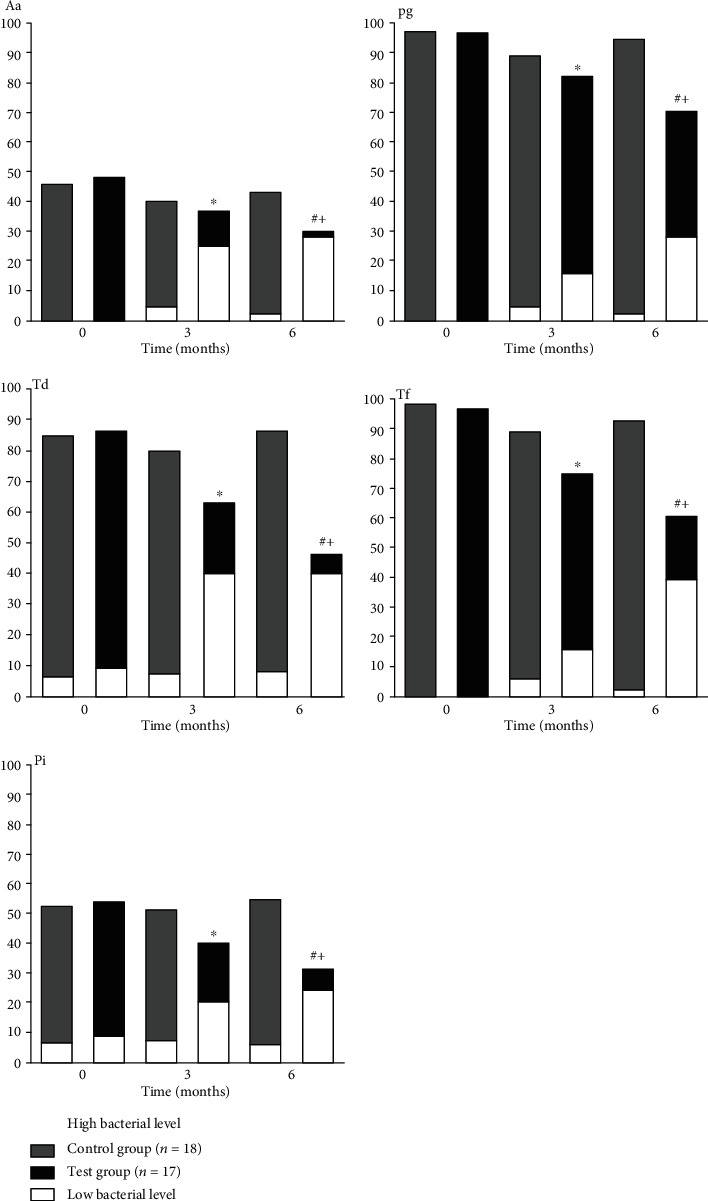
Proportions of patients (%) with low and high bacteria levels at the initial time point, 3 months, and 6 months of following up sorted by periopathogen and control versus test. ^∗^Within-group differences: 0.005 < *p* ≤ 0.05 (between initial time point and follow-up visits). ^+^Within-group differences: *p* ≤ 0.005. ^#^Between-group differences: 0.005 < *p* ≤ 0.05.

Figure 1 shows detection frequencies for each of the five periopathogenic species at different examination points per treatment strategy, taking low and high levels of bacteria into account. The results were expressed as the proportion of patients (%) in whom a given pathogen was detected at low levels or at high levels. A statistically significant decrease in the proportion of high-level sites of periopathogens was observed only in patients of the Test group after weekly supragingival irrigation with aerosolized 0.5% H_2_O_2_. Compared to the initial time point, in the Test group, there was a significant decrease in the proportion (%) of patients with high levels of Aa (*p* = 0.034), Pg (*p* = 0.018), Td (*p* = 0.05), Tf (*p* = 0.002), and Pi (*p* = 0.132) bacteria at 3 months and of Aa (*p* < 0.001), Pg (*p* = 0.001), Td (*p* < 0.001), Tf (*p* < 0.001), and Pi (*p* = 0.015) bacteria at 6 moths. As seen in the above-presented data, the proportion (%) of the Test group patients with high levels of bacteria continued to decrease after 3 months, but the findings did not differ essentially from those obtained after 6 months.

### 3.3. Data of Morphological Examination of Gingival Tissue

**Figure 2:**
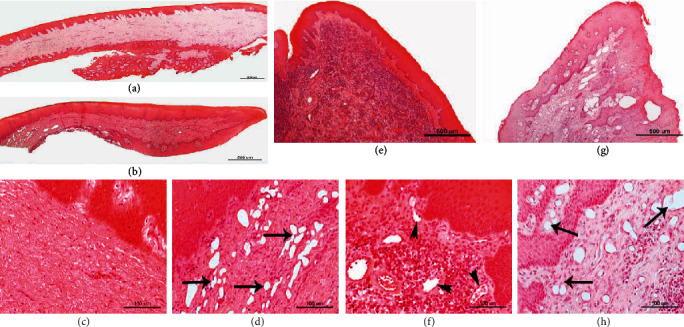
Microscopic study of the investigated human and porcine gingival tissues. (a, c) Healthy porcine gingival tissue with regular histological structure; (b, d) the investigated porcine gingival samples show cavities of irregular shape and size (arrows) in the connective tissue created after the irrigation procedures; (e, f) gingiva of periodontitis patients with prominent infiltration of inflammatory cells in the connective tissue beneath the epithelium; (g, h) irrigated gingiva of periodontitis patients shows spherical bubbles (arrows) in the connective tissue and a decreased infiltration of inflammatory cells. Arrowheads indicate blood vessels. Scale bars: (a, b, e, g) 500 *μ*m; (c, d, f, h) 100 *μ*m.

The morphological examination of diseased gingival tissues in patients with CP showed a changed extracellular matrix, damaged collagen fibers, and infiltration of multiple inflammatory cells (Figures 2(e) and 2(f)). The samples of the diseased gingival tissues obtained immediately after supplementary supragingival irrigation with aerosolized 0.5% H_2_O_2_ solution showed numerous spherical bubbles (Figures 2(g) and 2(h)). Bubble size varied greatly, most frequently reaching 15-50 *μ*m. In nonirrigated diseased gingival tissues of patients with chronic periodontitis and nonirrigated healthy gingival tissues of *ex vivo* porcine, no similar bubbles were observed (Figures 2(a) and 2(c)). It is necessary to note that the irrigated *ex vivo* porcine gingival tissues also showed numerous spherical bubbles (Figures 2(b) and 2(d)) similar to those mentioned above. The bubbles were round in shape, in some places interflowing into large irregular cavities.

## 4. Discussion

### 4.1. The Effect of Weekly Supragingival Irrigation as an Adjunct to NSPT as Compared to That of NSPT Alone on Clinical Parameters

The results of this study demonstrated significant benefits to oral health through a greater improvement in all the clinical parameters of oral hygiene and a reduction in gingival inflammation in patients of the Test group compared to the initial time point examination data and data of the Control group patients (Table 2). An eminent decrease in the API and BI indices in patients of the Test group was observed after 6 months of supportive care, which exceeded (*p* ≤ 0.01) the respective data of the Control group patients. H_2_O_2_ is known to be effective in reducing plaque [35]. The effectiveness of H_2_O_2_ in plaque removal has been attributed to the physical effect of the stream of H_2_O_2_ and to the bubbling of oxygen as it is released from peroxide [36]. Conceivably, our discovered cavitation bubbles in the gingival tissue played a role as well. Cavitation bubbles are known [37, 38] to be an effective agent in the surface treatment process. In addition to that, the oral hygiene of the patients of the Test group might also have improved. During the entire period of supportive care, the patients of the Test group maintained a good level of oral hygiene due to weekly visits to the dental office for irrigation procedures, where they were provided with additional information and were motivated regarding oral hygiene [30, 35, 39].

In this study, regular weekly supportive care of periodontitis patients of the Test group with aerosolized 0.5% H_2_O_2_ following NSPT prominently (*p* ≤ 0.05) decreased MGI, PPD, and the proportion (%) of periodontal sites showing PPD > 4 mm (Table 2). Meanwhile, NSPT alone had no influence on the aforementioned clinical indices in the Control group patients during the 6-month period. Our clinical results confirm the opinion of other authors [40, 41] who state that mechanical periodontal treatment alone results in a short-term reduction of periodontal inflammation. Armitage and Xenoudi [42] pointed out that long-term successful treatment of chronic periodontitis requires placement of these patients on posttreatment supportive periodontal therapy.

Our clinical data of regular supportive periodontal therapy (Test group) with weekly supragingival irrigation with aerosolized 0.5% H_2_O_2_ indirectly corroborate the data of Herrero et al. [17] that the amount of pathobionts in oral biofilms is suppressed by H_2_O_2_ and confirmed the aforementioned authors' statements that maintenance periodontal treatment is an essential component of long-term successful periodontal treatment. It is well known that periodontal pockets of periodontitis patients are contaminated with periopathogens, their toxins, and by-products of inflammation. Therefore, even irrigation with a sole set stream of water has a positive effect on subgingival microbiota due to the removal of aforementioned products from the gingival pockets [43–46]. In addition, supragingival irrigation has a positive effect on cytokine levels, which are associated with pathological changes in periodontal tissues [47, 48].

### 4.2. The Effect of Weekly Supragingival Irrigation as a Supportive Periodontal Therapy on the Microflora

The aim of the present study was also to investigate, using a multiplex PCR technique, the microbiological impact of regular weekly supragingival irrigation with aerosolized 0.5% H_2_O_2_ as an adjunct to root planning in patients with chronic periodontitis (Test group). This molecular method for bacterial identification seemed more advantageous than culturing because it is less labor-intensive, less time-consuming, and less expensive. The commercial microbiological kit used in this study had been validated some years before by Eick and Pfister [34] and had been shown to have high diagnostic sensitivity.

The results of the present study indicated that both treatment strategies resulted in microbial shifts at 3 months after treatment (Table 3). However, at the same time, the microbiological changes in patients of the Control group were without significant differences to the initial time point data and had a general tendency towards an increase at 6 months. This microbiological deterioration is the result of the proliferation of residual microbiota or subgingival recolonization as an extension of growing supragingival plaque. Essentially, there are three sources of residual microbiota after periodontal therapy: the periodontal pocket itself may act as a primary source following inadequate debridement. In addition, the surrounding hard and soft tissues may act as a bacterial reservoir from which recolonization and proliferation occur because some pathogenic species are capable of invading these tissues [49]. Our data showed that supportive periodontal therapy—regular weekly supragingival irrigation with aerosolized 0.5% H_2_O_2_—following NSPT resulted in marked microbiological shifts in the Test group (Table 3). The largest reduction was observed in the proportion (%) of sites positive for Pg, Td, and Tf. This probably was influenced by the improved clinical status of the sampled sites (the initial proportion of periodontal sites with PPD > 4 mm was 93% versus 60.2% after the treatment).

Periodontal disease is known to be significantly associated with the red complex species [50]. Out of the three members of the red complex, *P. gingivalis* and *T. denticola* have been strongly associated with the pathogenesis of periodontitis [51]. Our observation demonstrated (Figure 1) that weekly supragingival irrigation with aerosolized 0.5% H_2_O_2_ in the Test group provided a significant decrease in the proportions of patients with high levels of Aa (*p* = 0.034), Pg (*p* = 0.018), Td (*p* = 0.05), Tf (*p* = 0.001), and Pi (*p* = 0.132) at 3 months; at 6 months, there was a significant drop in the proportions of patients with high levels of Aa (*p* < 0.001), Pg (*p* = 0.001), Td (*p* < 0.001), Tf (*p* < 0.001), and Pi (*p* = 0.015). This observation is compatible with the greater clinical response in the Test group (Table 2) at 3 months and particularly at 6 months. These data corroborate the statement of Kumawat et al. [52] that increased levels of specific pathogens are needed for the progression of periodontal disease. Our data indicating a significant decrease in the proportions of sites with high levels of periopathogenic bacteria in periodontitis patients of the Test group could be associated with a significant decrease in the proportion of periodontal sites showing PPD > 4 mm in these patients (Table 2), which is in line with the data presented by many authors [50, 52, 53]. Our data are in concordance with the findings of other authors [54] indicating that periodontal pockets represent the location in which disease activity primarily takes place and that the use of additional chemical substances for nonsurgical periodontal treatment as a supportive periodontal therapy has led to new treatment strategies that help to reduce oral bacterial load and at the same time to control periodontal disease [55]. Conceivably, in our clinical investigation, this may have been influenced by many chemical products created during the collapse of cavitation bubbles in gingival tissues. Hence, regular visits during maintenance periodontal therapy—weekly supragingival irrigation with aerosolized 0.5% H_2_O_2_ solution—positively influenced subgingival microbiota and contributed to the improvement of the periodontal clinical status in patients with chronic periodontitis. Thus, the results from this randomized clinical trial confirm our previous data [14] on the efficacy of supragingival irrigation with the aerosolized 0.5% H_2_O_2_ solution as a maintenance periodontal therapy on the clinical parameters of periodontitis patients and confirm our described phenomenon of formation of cavitation bubbles not only in periodontitis-damaged gingival tissues but also in experimental *ex vivo* porcine healthy gingival tissues as a consequence of these irrigation.

### 4.3. The Formation of Cavitation Bubbles in Periodontal Tissues

In this study, as in our recent work [14], we observe the formation of numerous thin-walled spherical bubbles in diseased gingival tissues of patients with CP (Figures 2(g) and 2(h)) under the influence of irrigation with the aerosolized 0.5% H_2_O_2_ solution. However, this time our aim was to investigate whether irrigation causes the same result in *ex vivo* porcine healthy gingival tissues. And we found the same formation of abundant spherical bubbles in *ex vivo* porcine healthy gingival tissues after irrigation procedure with the aerosolized 0.5% H_2_O_2_ solution (Figures 2(b) and 2(d)). These bubbles have many similarities to cavitation bubbles that form following acoustic cavitation [20]. The size of the bubbles was 10-50 *μ*m. They were round in shape, in some places merging and forming large cavities up to 100 *μ*m and more (Figures 2(b), 2(d), 2(g), and 2(h)). No such changes were observed in gingival tissue samples of periodontitis patients (Figures 2(e) and 2(f)) and healthy porcine (Figures 2(a) and 2(c)), which have not undergone irrigation procedure.

As we have not found similar data in the literature, we continue to hold the view that spherical bubbles formed as a result of irrigation with aerosolized H_2_O_2_ solution are probably cavitation bubbles. The size of the bubbles we discovered in gingival tissues corresponds to the description of cavitation bubbles in the literature [56]. It is unclear how the cavitation bubbles penetrated into the deeper layers of the gingival tissue because no damage to the intercellular matrix of the gingival tissues was seen. (Figures 2(d) and 2(h)). Several studies have conclusively shown that ultrasound-induced enhanced skin permeabilization is mediated by acoustic cavitation [21, 57]. Cavitation is a complex phenomenon that involves formation, oscillation, growth, and collapse of bubbles within a liquid medium depending on local pressure variation [27]. Complex chemical reactions take place during the bubble collapse, and many reactive oxygen species can be generated [25], which, in turn, may form a bactericidal medium in deeper layers of the tissues. We believe that this characteristic can be highly important in the treatment of periodontal diseases. Moreover, the hydrogen formed during these reactions can selectively neutralize the ROS and attenuate the associated inflammatory reactions [58]. Collapse of the bubbles releases great amounts of energy and may cause damage to exposed surfaces. However, Itah et al. [59] in their study found that the application of cavitation did not lead to prominent DNA damage, cell cycle arrest, or the initiation of programmed cell death (i.e., classical apoptosis or autophagy activation). Natural hydrodynamic cavitation occurs in liquids when the pressure drops below vapor pressure. This creates cavities and cavitation bubbles in liquid filled with steam and diffused gases [60]. The bubble collapse hypothesis not only is intuitively clear because released shock waves easily penetrate and create pores in the membrane but has also been confirmed by both experiment and theory [61]. Hence, a single bubble has attracted much attention owing to both the apparent simplicity of its formation and the complexity of the chemical reactions occurring within it [62]. The use of the cavitation phenomenon in medicine is widely discussed [63], especially for the introduction of drugs into selectively permeable tissues [64, 65]. It is mentioned in the medical literature that cavitation bubbles can be used to transport drugs to deeper tissues [66–68]. It should be clarified whether cavitation bubbles caused by irrigation could perform such functions, because this would be very useful in the treatment of periodontitis.

### 4.4. Trial Limitations and Outlook in the Future

Our study has a few limitations: small study groups, short study time, and inadequate Control group. The data do not allow a separate assessment of the influence of aerosolized hydrogen peroxide, irrigation stream, or cavitation bubbles on the above-mentioned parameters. The data from this study present only the overall effect of aerosolized hydrogen peroxide solution, irrigation stream, and formation of cavitation bubbles on changes in these parameters.

However, data from this work suggest that long-term regular supragingival irrigation with aerosolized 0.5% hydrogen peroxide has a positive effect on clinical and microbiological parameters in patients with periodontitis. Additional studies are needed to evaluate the significance of separate impact of aerosolized hydrogen peroxide, irrigation stream, and cavitation bubbles in plaque reduction. It is appropriate to investigate whether hydrodynamic cavitation bubbles can be used to track the drug carrier to become entrapped in microbubbles in selectively permeable regions. This would allow the development of more accurate and less expensive methods for the treatment and prevention of periodontal disease.

## 5. Conclusions

1. The effectiveness of mechanical periodontal treatment combined with weekly supragingival irrigation with aerosolized 0.5% H_2_O_2_ solution on clinical and microbiological parameters of periodontal tissues of periodontitis patients is reliably higher than mechanical periodontal debridement alone
2. It has been found that cavitation bubbles as a result of irrigation with the aerosolized 0.5% hydrogen peroxide solution can form not only in periodontal tissues of periodontitis patients but also in *ex vivo* porcine healthy gingival tissues
3. Further investigations are needed to fully explore the potential effects of irrigation with the aerosolized hydrogen peroxide solution and to research into the phenomenon of hydrodynamic cavitation bubbles (as a result of irrigation with the aerosolized hydrogen peroxide solution) and its possible application in the supportive therapy of periodontitis and/or drug delivery to certain tissues

## 6. Clinical Relevance

Regular visits during preventive maintenance therapy with weekly supragingival irrigation with aerosolized 0.5% hydrogen peroxide solution and the formation of cavitation bubbles in gingival tissues positively influenced subgingival microbiota and contributed to the improvement of the clinical status of patients with chronic periodontitis. Thus, this technique (particularly for its low cost) may be recommended as an additional oral hygiene procedure for supportive periodontal therapy.

## Figures and Tables

**Table 1:** Initial time point data of the patients (*M* ± SD) and distribution of sampled sites.

	Control group	Test group	Independent sample test
Age	43.3 ± 5.6	44.2 ± 6.1	NS
Gender (M/F)	8/10	8/9	NS
API (%)	36.1 ± 4.2	35.6 ± 4.1	NS
MGI (points)	2.6 ± 0.5	2.7 ± 0.6	NS
BI (%)	68.9 ± 5.5	69.8 ± 5.3	NS
PD (mm)	5.3 ± 0.6	5.4 ± 0.6	NS
Distribution of sampled sites			
Singled-rooted teeth (*n*)	39	36	NS
Multirooted teeth (*n*)	33	32	NS
Furcation involved sites (*n*)	13	14	NS
Nonfurcation involved sites (*n*)	59	54	NS

NS: not significant.

**Table 2:** Clinical data of sampled sites (*M* ± SD) at different time points.

Time points	Treatment groups	Periodontal parameters				
		API (%)	MGI (points)	BI (%)	PPD (mm)	Percentage of sites showing PPD > 4 mm
Initial	Control (*n* = 18)	36.1 ± 4.2	2.6 ± 0.5	68.9 ± 5.5	5.3 ± 0.6	93
	Test (*n* = 17)	35.6 ± 4.1	2.7 ± 0.6	69.8 ± 5.3	5.4 ± 0.6	95
3 months	Control (*n* = 18)	30.3 ± 3.1^∗^	2.1 ± 0.4	34.2 ± 4.1^#^	4.7 ± 0.7	89.5
	Test (*n* = 17)	22.2 ± 2.6^#^	1.8 ± 0.2^∗^	21.5 ± 5.8^#$\bar{⊤}$^	4.0 ± 0.6	75.6
6 months	Control (*n* = 18)	29.0 ± 2.7^∗^	2.2 ± 0.3	31.5 ± 4.2^#^	4.9 ± 0.6	91.7
	Test (*n* = 17)	12.4 ± 2.3^∗∗^^&^	1.3 ± 0.2^∗^^$\bar{⊤}$^	12.2 ± 3.6^∗∗^^&^	3.2 ± 0.7^∗^	60.2^∗^^$\bar{⊤}$^

API: Approximal Plaque Index; MGI: Modified Gingival Index; BI: Bleeding Index; PPD: Pocket Probing Depth. ^∗^Within-group differences: *p* ≤ 0.05 (between initial time point and follow-up visits). ^∗∗^Within-group differences: *p* ≤ 0.05 (between 3 months and follow-up visits). ^#^Within-group differences: *p* ≤ 0.01 (between initial time point and follow-up visits). ^$\bar{⊤}$^Between-group differences: *p* ≤ 0.05. ^&^Between-group differences: *p* ≤ 0.01.

**Table 3:** Detection frequencies sorted by periopathogen (%).

Periopathogen	Treated groups	Initial time point	Month 3	Month 6
Aa	Control (*n* = 18)	46	39	43
	Test (*n* = 17)	48	37	30
Pg	Control (*n* = 18)	98	90	95
	Test (*n* = 17)	97	83	71^∗^^#^
Pi	Control (*n* = 18)	53	48	56
	Test (*n* = 17)	55	41	32
Td	Control (*n* = 18)	85	80	86
	Test (*n* = 17)	86	63	46^∗^^#^
Tf	Control (*n* = 18)	100	90	94
	Test (*n* = 17)	98	76	61^∗^^#^

^∗^Within-group difference: *p* ≤ 0.05 (between initial time point and follow-up visit). ^#^Between-group difference: *p* ≤ 0.05.

## Data Availability

The data used to support the findings of this study are available from the corresponding author upon request.
